# The *Caenorhabditis elegans* Dispatched ortholog, CHE-14, is dispensable for apical secretion of the Hedgehog-related proteins GRL-2 and WRT-10

**DOI:** 10.17912/micropub.biology.001329

**Published:** 2024-09-25

**Authors:** Nicholas D Serra, Meera V Sundaram

**Affiliations:** 1 Dept. of Genetics, Perelman School of Medicine, University of Pennsylvania, Philadelphia, Pennsylvania, United States

## Abstract

*C. elegans* nematodes possess expanded families of Hedgehog related (Hh-r) and Patched/Dispatched-related (PTR) proteins but their functional relationship remains unclear. Here we investigated whether CHE-14 , the closest *C. elegans* ortholog for the Hedgehog transporter Dispatched, was necessary for the secretion of two tagged Hh-r proteins: WRT-10 and GRL-2 . We report that CHE-14 is dispensable for the apical localization of GRL-2 and WRT-10 . We also show that animals lacking CHE-14 and another redundant PTR protein DAF-6 also secrete WRT-10 , suggesting neither are required for secretion of these specific Hh-r proteins.

**Figure 1. Hh-r proteins GRL-2 and WRT-10 localize normally in che-14 mutant nematodes:**
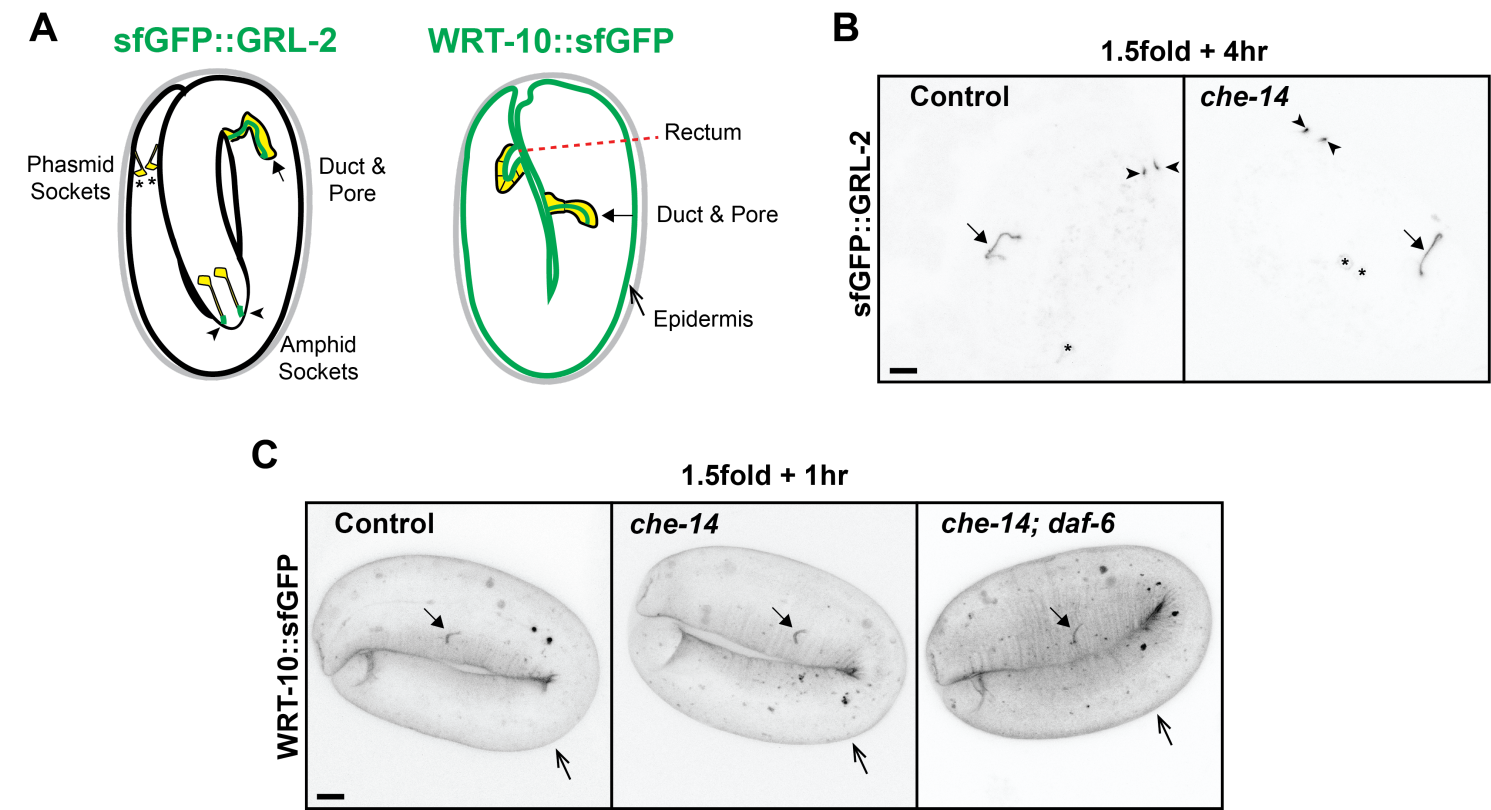
A) Schematic diagrams of Hh-r fusion protein localization (green) in embryos. The cytoplasm of cells of interest is indicated in yellow. sfGFP::GRL-2 localizes to the lumens of specific unicellular tubes, the amphid glia socket cells and the excretory duct and pore. sfGFP::GRL-2 is observed cytoplasmically in the phasmid socket glia, and localizes to this cell's lumen later in development (Serra et al., 2024). WRT-10::sfGFP localizes to the epidermal surface, the rectum, and the excretory duct and pore lumens. Stages and symbols match those in the micrographs below. B) sfGFP::GRL-2 localizes to the cuticle of the excretory duct (arrow) and amphid socket lumens (arrowhead) in both *che-14* and control animals. Asterisks mark the cytoplasmic expression in phasmid socket cells. C) WRT-10::sfGFP localizes to the embryonic sheath matrix lining the epidermis (open arrow) and excretory duct and pore (closed arrow) in *che-14, che-14* ; *daf-6* double mutants, and control animals. Images shown are max projections of full-embryo z-stacks. In all panels, scale bars= 5 µm. At least n=10 animals were observed for each genotype. Embryos were obtained from homozygous mutant mothers of the indicated genotype, except in double mutant animals the mothers contained a rescue transgene ( *csEx946* ). Additional characterization of these fusion proteins in wild-type animals can be found in (Serra et al., 2024).

## Description

Despite lacking clear orthologs for the Hedgehog signaling ligand and the majority of its canonical pathway partners, *Caenorhabditis elegans* nematodes possess 26 Patched/Dispatched-related (PTR) proteins (the canonical Hedgehog receptor and transporter, respectively) and over twice as many small proteins considered to be evolutionarily Hedgehog-related (Hh-r) (Bürglin and Kuwabara 2006; Bürglin 2008; Hao et al., 2006; Zugasti et al., 2005; Aspöck et al., 1999) . We recently designed endogenously tagged sfGFP fusion proteins for two Hh-r proteins, GRL-2 and WRT-10 , and found that they were both tissue and substructure specific apical extracellular matrix (aECM) components (Serra et al., 2024) . GRL-2 , which belongs to the Groundhog-like (GRL) family, is a component of specific tube cuticles, while WRT-10 , which belongs to the Warthog (WRT) family, is a component of the transient precuticle or "sheath" matrix that precedes the cuticle in developing worms ( Figure 1A ). Other Hh-r proteins, such as GRL-7 and GRL-18 , have also been localized to specific aECMs (Chiyoda et al., 2021; Fung et al., 2023) . Many PTR proteins also affect the aECM (reviewed Sundaram and Pujol 2024), but the specific relationships between Hh-r and PTR proteins remain unclear.

In canonical Hedgehog signaling, Dispatched is a necessary mediator of Hedgehog trafficking and release (reviewed in Zhang and Beachy 2023). Among *C. elegans* proteins, the PTR CHE-14 shares the most sequence homology with Dispatched (Michaux et al., 2000; Bürglin and Kuwabara 2006) . Furthermore, CHE-14 has previously reported roles in apical secretion to promote proper aECM organization in the epidermis, excretory system tubes, and amphid glia (Michaux et al., 2000; Perens and Shaham 2005) . Therefore, we hypothesized that CHE-14 might promote the apical secretion of Hh-r proteins from these tissues.

Here we show that, counter to our hypothesis, both GRL-2 and WRT-10 sfGFP fusion proteins are still properly secreted and localize to matrix in *che-14* ( *ok193* ) null mutant animals. In both wild-type and *che-14* mutant embryos, GRL-2 localizes to the cuticle matrix lining the amphid socket and the excretory duct and pore lumens ( Figure 1B ). WRT-10 also localizes to the apical matrix of the epidermis, the rectum, and the excretory duct and pore in both wild type and mutant embryos ( Figure 1C ). Although a mild effect could be missed in these qualitative analyses, these observations indicate that CHE-14 is not necessary for the secretion and apical localization of GRL-2 and WRT-10 .

Another *ptr* gene, *daf-6* , has been reported to function redundantly with *che-14* in single-cell tubulogenesis, and loss of function of both genes causes synthetic larval lethality (Perens and Shaham 2005) . However, embryos mutant for both genes were still able to secrete WRT-10 ( Figure 1C ). Given the large number of *ptr* genes in *C. elegans* , it is possible that other *ptr* genes expressed in these epithelia act redundantly with *che-14* to transport GRL-2 and WRT-10 , or that a different *ptr* gene is specifically required. It is also possible that CHE-14 transports different Hh-r proteins than those studied here, or perhaps *C. elegans* Hh-r proteins may not rely on PTR proteins for secretion.

A recent report demonstrated that endocytosis of the Hh-r GRL-7 specifically requires PTR-18 , suggesting that the PTR-proteins might regulate endocytosis or other aspects of Hh-r protein trafficking (Chiyoda et al., 2021) . Emerging data also suggest that some Hh-r proteins work with specific PTR proteins in possible signaling roles; however, the pathways that connect them with downstream effects remain elusive (Riveiro et al., 2017; Lin and Wang 2017; Kume et al., 2019; Templeman et al., 2020; Chiyoda et al., 2021; Shi and Murphy 2023; Wang et al., 2023; Emans et al., 2023) . Further work is needed to reveal the connection between these protein families in nematodes.

## Methods

*C. elegans* culture and maintenance:

All strains were maintained at 20˚ C on nematode growth medium (NGM) seeded with OP50 *E. coli* under standard conditions (Brenner 1974) . Information from Wormbase was referenced for all experimental design (Sternberg et al., 2024) . Strains used are listed in the Reagent table.

Confocal imaging of fluorescent proteins in elongating *C. elegans* embryos:

Images were captured using a Leica TCS DMi8 confocal microscope and Leica Application Suite X software (version 3.5.7.23225). This microscope was equipped with an HC PL APO 63X objective lens (Numerical Aperture 1.3). Entire embryos were imaged by collecting a series of 60-75, 0.33 µm Z-slices. SfGFP fusions were visualized using a 488 nm laser set to 3% power and emitted wavelengths between 493 and 578 nm were collected at a scanning speed of 700 hz with a HyD sensor. *C. elegans* embryos were picked at the 1.5fold stage of development and incubated at 20˚ C for the indicated number of hours prior to imaging. Samples were mounted with 10 mm levamisole in M9 buffer on pads of 2% agar noble and 2.5% sodium azide.

## Reagents

**Table:** 

**Strain**	**Genotype**	**Source**
ML514	*che-14* ( *ok193* ) I	CGC, Michaux et al., 2000
CB1377	*daf-6* ( *e1377* ) X	CGC, Perens and Shaham 2005
PHX7022	*wrt-10* ( *syb7022* [ WRT-10 ::sfGFP]) II	SunyBiotech, Serra et al., 2024
PHX7064	*grl-2* ( *syb7064* [ssSfGFP:: GRL-2 ]) V	SunyBiotech, Serra et al., 2024
UP4228	*che-14* ( *ok193* ) I; *wrt-10* ( *syb7022* ) II	This work
UP4229	*che-14* ( *ok193* ) I; *grl-2* ( *syb7064* ) V	This work
UP4274	*che-14 ( ok193 ) I; wrt-10 (syb7022) II; daf-6 ( e1377 ) X; csEx946( che-14 +; unc-119p::GFP) *	This work. The *che-14* rescue transgene *csEx946* was generated by co-injecting fosmid WRM0620dD06 [20 ng/µl] and pIM175 (unc-119p::GFP) [100 ng/µl].

## References

[R1] Aspöck G, Kagoshima H, Niklaus G, Bürglin TR (1999). Caenorhabditis elegans has scores of hedgehog-related genes: sequence and expression analysis.. Genome Res.

[R2] Brenner S (1974). The genetics of Caenorhabditis elegans.. Genetics.

[R3] Bürglin TR, Kuwabara PE (2006). Homologs of the Hh signalling network in C. elegans.. WormBook.

[R4] Bürglin TR (2008). Evolution of hedgehog and hedgehog-related genes, their origin from Hog proteins in ancestral eukaryotes and discovery of a novel Hint motif.. BMC Genomics.

[R5] Chiyoda H, Kume M, Del Castillo CC, Kontani K, Spang A, Katada T, Fukuyama M (2021). Caenorhabditis elegans PTR/PTCHD PTR-18 promotes the clearance of extracellular hedgehog-related protein via endocytosis.. PLoS Genet.

[R6] Emans SW, Yerevanian A, Ahsan FM, Rotti JF, Zhou Y, Cedillo L, Soukas AA (2023). GRD-1/PTR-11, the C. elegans hedgehog/patched-like morphogen-receptor pair, modulates developmental rate.. Development.

[R7] Fung W, Tan TM, Kolotuev I, Heiman MG (2023). A sex-specific switch in a single glial cell patterns the apical extracellular matrix.. Curr Biol.

[R8] Hao L, Johnsen R, Lauter G, Baillie D, Bürglin TR (2006). Comprehensive analysis of gene expression patterns of hedgehog-related genes.. BMC Genomics.

[R9] Kume M, Chiyoda H, Kontani K, Katada T, Fukuyama M (2019). Hedgehog-related genes regulate reactivation of quiescent neural progenitors in Caenorhabditis elegans.. Biochem Biophys Res Commun.

[R10] Lin CJ, Wang MC (2017). Microbial metabolites regulate host lipid metabolism through NR5A-Hedgehog signalling.. Nat Cell Biol.

[R11] Michaux G, Gansmuller A, Hindelang C, Labouesse M (2000). CHE-14, a protein with a sterol-sensing domain, is required for apical sorting in C. elegans ectodermal epithelial cells.. Curr Biol.

[R12] Perens EA, Shaham S (2005). C. elegans daf-6 encodes a patched-related protein required for lumen formation.. Dev Cell.

[R13] Riveiro AR, Mariani L, Malmberg E, Amendola PG, Peltonen J, Wong G, Salcini AE (2017). JMJD-1.2/PHF8 controls axon guidance by regulating Hedgehog-like signaling.. Development.

[R14] Serra ND, Darwin CB, Sundaram MV (2024). Caenorhabditis elegans Hedgehog-related proteins are tissue- and substructure-specific components of the cuticle and precuticle.. Genetics.

[R15] Shi C, Murphy CT (2023). piRNAs regulate a Hedgehog germline-to-soma pro-aging signal.. Nat Aging.

[R16] Sternberg PW, Van Auken K, Wang Q, Wright A, Yook K, Zarowiecki M, Arnaboldi V, Becerra A, Brown S, Cain S, Chan J, Chen WJ, Cho J, Davis P, Diamantakis S, Dyer S, Grigoriadis D, Grove CA, Harris T, Howe K, Kishore R, Lee R, Longden I, Luypaert M, Müller HM, Nuin P, Quinton-Tulloch M, Raciti D, Schedl T, Schindelman G, Stein L (2024). WormBase 2024: status and transitioning to Alliance infrastructure.. Genetics.

[R17] Sundaram MV, Pujol N (2024). The Caenorhabditis elegans cuticle and precuticle: a model for studying dynamic apical extracellular matrices in vivo.. Genetics.

[R18] Templeman NM, Cota V, Keyes W, Kaletsky R, Murphy CT (2020). CREB Non-autonomously Controls Reproductive Aging through Hedgehog/Patched Signaling.. Dev Cell.

[R19] Wang Q, Fu R, Li G, Xiong S, Zhu Y, Zhang H (2023). Hedgehog receptors exert immune-surveillance roles in the epidermis across species.. Cell Rep.

[R20] Zhang Y, Beachy PA (2023). Cellular and molecular mechanisms of Hedgehog signalling.. Nat Rev Mol Cell Biol.

[R21] Zugasti O, Rajan J, Kuwabara PE (2005). The function and expansion of the Patched- and Hedgehog-related homologs in C. elegans.. Genome Res.

